# Chronic Diarrhea Caused by Vasoactive Intestinal Peptide-Secreting Tumor

**DOI:** 10.3390/life13101974

**Published:** 2023-09-27

**Authors:** Oana Belei, Diana-Georgiana Basaca, Elena Rodica Heredea, Emil Radu Iacob, Laura Olariu, Roxana Folescu, Andrei Gheorghe Marius Motoc, Anda-Maria Nanu, Otilia Mărginean

**Affiliations:** 1 First Pediatric Clinic, Disturbances of Growth and Development on Children Research Center, “Victor Babeș” University of Medicine and Pharmacy, 300041 Timișoara, Romania; belei.oana@umft.ro (O.B.); marginean.otilia@umft.ro (O.M.); 2 Third Pediatric Clinic, “Louis Țurcanu” Emergency Hospital for Children, 300011 Timișoara, Romania; tunealaura@yahoo.com (L.O.); anda.nanu97@yahoo.com (A.-M.N.); 3 Department of Pathology, “Louis Ţurcanu” Emergency Hospital for Children, 300011 Timișoara, Romania; heredea.rodica@yahoo.com; 4 Department of Clinical Practical Skills, “Victor Babeş” University of Medicine and Pharmacy, 300041 Timişoara, Romania; 5 Department of Pediatric Surgery, “Victor Babeș” University of Medicine and Pharmacy, 300041 Timișoara, Romania; radueiacob@umft.ro; 6 First Pediatric Clinic, “Victor Babeș” University of Medicine and Pharmacy, 300041 Timișoara, Romania; 7 Department of Balneology, Medical Recovery, and Rheumatology, Family Medicine Discipline, Center for Preventive Medicine, “Victor Babeș” University of Medicine and Pharmacy, 300041 Timișoara, Romania; roxanafolescu08@gmail.com; 8 Department of Anatomy and Embryology, “Victor Babeș” University of Medicine and Pharmacy, 300041 Timișoara, Romania; amotoc@umft.ro

**Keywords:** infant, diarrhea, vasoactive intestinal polypeptide, ganglioneuroblastoma

## Abstract

VIPomas are a type of neuroendocrine tumor that independently produces vasoactive intestinal peptide (VIP). VIPomas causing watery diarrhea, hypokalemia, and achlorhydria (WDHA) syndrome are not frequently observed in adult patients without pancreatic ailments. However, in children, the occurrence of a VIPoma originating in the pancreas is exceedingly uncommon. Instead, WDHA syndrome is more commonly associated with neurogenic tumors that secrete VIP, often located in the retroperitoneum or mediastinum. Among infants, chronic diarrhea is a prevalent issue that often necessitates the attention of pediatric gastroenterologists. The underlying causes are diverse, and delays in arriving at a definitive diagnosis can give rise to complications affecting the overall well-being of the child. The authors present the case of an infant with chronic watery diarrhea, subocclusion manifestations, mild hypokalemia, and metabolic hyperchloremic acidosis secondary to a VIPoma in the retroperitoneum that was diagnosed via abdominal ultrasound and tomography. The laboratory results revealed lowered potassium levels and an excessive secretion of VIP. Following the surgical removal of the tumor, the diarrhea resolved, and both electrolyte levels and the imbalanced hormone levels returned to normal. Immunohistochemical examination confirmed the diagnosis of ganglioneuroblastoma, with N-MYC negative on molecular biology tests. We present the clinical and histo-genetic aspects of this rare clinical entity, with a literature review.

## 1. Introduction

In 1958, Verner and Morrison initially documented the occurrence of intractable aqueous diarrhea and a deficiency in potassium levels (hypokalemia), concomitant with noninsulin-secreting neoplasms originating from the islets of the pancreas [1]. Gastric hypersecretion was notably absent, and instances of achlorhydria were observed in individuals affected by this tumor syndrome, subsequently labeled as “pancreatic cholera” due to the presence of profound diarrhea, reminiscent of the clinical presentation seen in Vibrio cholerae infection [2]. The abbreviation WDHA (watery diarrhea, hypokalemia, achlorhydria) was suggested, although a potentially more fitting acronym could be WDHHA, representing watery diarrhea, hypokalemia, hypochlorhydria, and acidosis, taking into account the excess bicarbonate loss [3]. In addition to the classic symptoms of VIPoma, other presentations may also occur. These can include hyperglycemia, flushing, abdominal distension that is not in proportion to the patient’s nutritional state, and growth arrest [4]. The correlation between specific pancreatic cancers and watery diarrhea syndrome has been proven by several published series [5,6,7]. Although nonpancreatic tumors, such as bronchogenic carcinoma, medullary thyroid carcinoma, retroperitoneal histiocytoma, and adrenal pheochromocytoma, are rarely the cause of this disease in adults, they are most frequently linked to pancreatic islet cell tumors [8]. It is highly uncommon for a VIP-producing tumor to develop in the pancreas in babies; instead, WDHA syndrome is typically linked to VIP-secreting neurogenic tumors involving the mediastinum and retroperitoneum [6]. VIP is an endogenous that is produced at a physiological rate and concentration and is involved in several processes, including controlling gastrointestinal secretions [9]. VIP is expressed in the neurons of the respiratory, urogenital, and digestive systems in addition to the central nervous system, specifically the cortex, hippocampus, and hypothalamus [9]. The hormone acts as a powerful adenylate cyclase stimulator, causing the intestinal mucosa to secrete water and electrolytes. Adenosine 3′,5′-cyclic phosphate synthesis in the gut is stimulated by the hormone VIP, among its other functions cyclic adenosine monophosphate (cAMP). Increased cAMP levels brought on by excessive VIP secretion in VIPomas cause copious diarrhea and a significant loss of water and electrolytes (pancreatic cholera) [10]. An uncommon neoplasm known as a VIP tumor causes severe morbidity and mortality by secreting water and electrolytes into the gastrointestinal tract [9].

Pancreatic non-beta-cell hyperplasia is a rare condition, although it is observed in youngsters. The majority of medical training is based on case reports [11,12]. Currently, the incidence of this disease among the pediatric population is unclear but, according to the latest studies, the annual incidence would be about 1 in 10 million people [13].

Pediatric patients may experience persistent diarrhea due to a wide variety of gastrointestinal disorders. Secretory diarrhea, which occurs in a tiny proportion of patients, is caused by active intraluminal fluid secretion. The identification of such pathologic cases is essential due to their significant diagnostic implications. A hidden VIPoma, a tumor that secretes VIP, may be the most likely cause of persistent secretory diarrhea. In children, extrapancreatic lesions are predominant; they are represented by pheochromocytoma, ganglioneuroblastoma, ganglioneuroma, and mastocytoma [14]. The diarrhea associated with VIPoma is typically chronic and often unresponsive to medical treatment. It is also profuse, with patients experiencing a significant loss of fluids (6–8 liters per day). Importantly, the diarrhea may persist, even under fasting conditions.

The diarrhea is characterized by an extended duration of watery consistency, with a fasting stool volume exceeding 750–1000 mL/day. It is not septic and typically begins slowly, with a gradual increase in frequency over several years. The episodes of diarrhea become more persistent, occurring more than 10 times per day. If the diarrhea becomes severe, it can cause dehydration and an uncontrollable imbalance in electrolytes and acid levels, leading to acidosis. The hyperchlorhydria previously described is exacerbated by the inhibitory impact of VIP on parietal gastric mucosal cells, which potentiates dietary magnesium, zinc, and vitamins C, K, and B deficits linked to malabsorption [15]. Skin rash, bloating, nausea, vomiting, tiredness, and an uncontrollable loss of weight are additional VIPoma symptoms [16]. Additionally, reports of hypomagnesemia with tetany brought on by diarrhea have been documented. In addition, VIP causes hyperglycemia (20–50%) by having a glycogenolysis impact on the liver [17]. However, continuous VIP infusion in humans results in tachyphylaxis, which could account for why so few individuals experience flushing.

While the majority of these tumors cannot be removed completely, a multifaceted strategy can control both the symptoms and the tumor, allowing most patients to live a long life. As a result of the rarity of this neoplasm and the paucity of prospective data, there are no precise guidelines for the management of VIPomas [15].

Healthcare providers need to recognize these additional symptoms when evaluating patients with VIPoma. Early diagnosis and treatment of VIPoma can help alleviate symptoms and prevent potentially life-threatening complications [18].

## 2. Objectives of the Study

The authors of this report describe a child diagnosed with chronic watery diarrhea and hypokalemia due to a retroperitoneal VIP-producing tumor, which immunohistochemical and molecular biology tests confirmed to be N-MYC-negative ganglioneuroblastoma. The symptoms were entirely resolved, and the preoperatively increased plasma VIP level returned to normal following surgical removal of the tumor. The case report is followed by a narrative review of the literature on this topic.

## 3. Case Presentation

A female patient, aged 11 months, was directed to our hospital due to a past of watery diarrhea, malnutrition, and significant abdominal distension. Vomiting was not reported. Over a month, she encountered prolonged bouts of watery diarrhea, occurring as frequently as 7–8 times a day, with no presence of blood or mucus.

The mother, who is 42 years old, and the father, who is 40 years old, are both from a rural area.

There was no evidence of a relevant family history of chronic illnesses. The female infant, who was the eighth to be delivered naturally, had a gestational age of 38 weeks, weighed 3100 g, and received a score of 9 on the APGAR scale. According to the national program, the child received his or her vaccinations. During the first month of life, the infant was naturally fed with her mother’s milk and then she received a milk formula. At the age of four months, supplemental feeding was also properly started. There is no relevant medical history for the infant. Her digestive function was normal for the first 10 months of her life. However, watery diarrhea started at the age of 10 months without a fever or vomiting.

Before being admitted to the hospital, a diagnosis of a protein from cow’s milk allergy was made after four weeks of outpatient testing. The infant commenced a lactose-free, protein-hydrolysate formula meal, but the trial of cow’s milk exclusion was unsuccessful. The initial antiparasitic treatment for unproved giardiasis was ineffective. Despite the administration of oral rehydration solution on multiple occasions, the infant’s diarrhea endured without resolution.

The pediatric patient was admitted with a stature measuring 70 cm, corresponding to the 5th percentile for their age, and a weight of 8 kg, aligning with the 25th percentile for their age. A physical examination revealed a regular heart rate of 85 beats per minute and normal blood pressure of 80/55 mmHg. The baby had significant abdominal distension, facial flushing, and no palpable abdominal mass or enlargement of the liver or spleen. Laboratory testing revealed mild metabolic acidosis and hypokalemia. The outcomes of a comprehensive analysis of blood cell counts and standard biochemical assessments fell within established normal parameters. We excluded the presence of infectious enterocolitis, intestinal parasitosis, celiac disease, exocrine pancreas insufficiency/mucoviscidosis, and cow’s milk protein allergy. The diarrhea persisted despite the introduction of a modular, elemental amino-acid-based dietary regimen.

**Figure 1:**
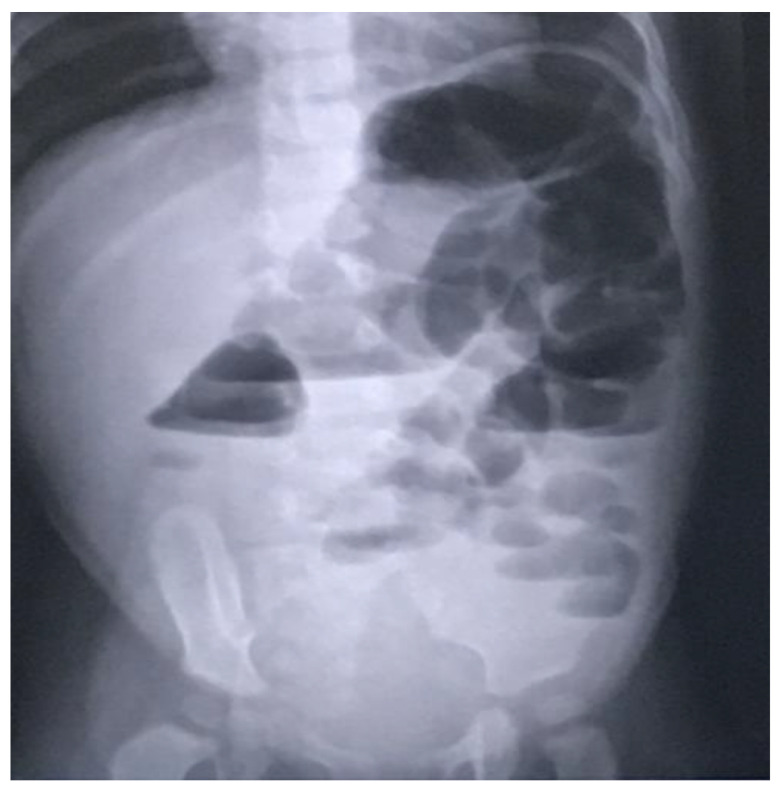
Radiographic indications of bowel distension with discernible air-fluid levels and the nonexistence of any lesion causing luminal occlusion.

In light of the ongoing significant abdominal distension, an abdominal X-ray was conducted, revealing bowel dilation characterized by air-fluid levels and the absence of any luminal obstructive lesion (Figure 1).

**Figure 2:**
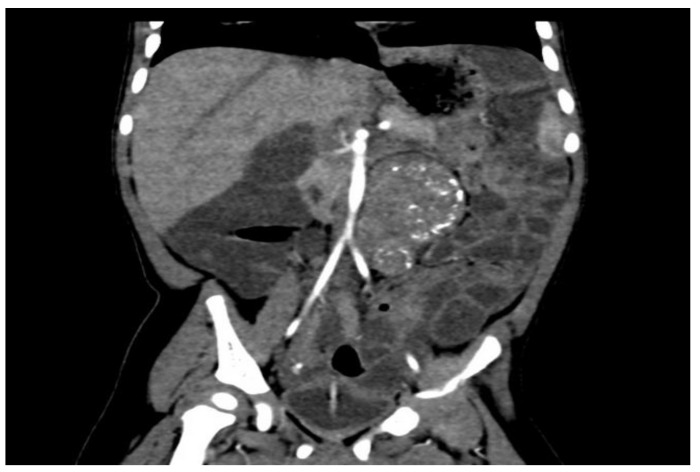
Retroperitoneal tumor with calcifications, as shown on a CT scan.

Following an abdominal computed tomography (CT) scan, an abdominal ultrasound revealed a calcified mass approximately 6/4 cm in diameter anterior to the L1–L4 lumbar spine and aorta but not in contact with the left kidney. There were no anomalies in the pancreas (Figure 2).

A further endocrinological assessment was conducted to obtain a precise diagnosis. There were no symptoms of thyroid dysfunction. When plasma hormone levels were examined, it was found that VIP concentrations were increased (normal range: 30 pmol/L; VIP = 180 pmol/L). Additionally, the levels of homovanillic acid and vanillylmandelic acid in the 24 urinary samples were both normal. However, increased levels of neuron-specific enolase (normal range: 16.3–25.77 ng/ml) raised concerns about a potential VIP-secreting ganglioneuroblastoma. The Department of Pediatric Surgery received a referral regarding the infant. A 6/4 cm encapsulated retroperitoneal tumor without contact with the left kidney was discovered during surgical exploration, anterior to the L1–L4 lumbar spine and aorta. There were no obvious alterations in the pancreas’ appearance. The next step was to remove the tumor, after which a slide was stained using the hematoxylin and eosin method for histopathologic analysis, and additional immunohistochemical and molecular tests were carried out.

**Figure 3:**
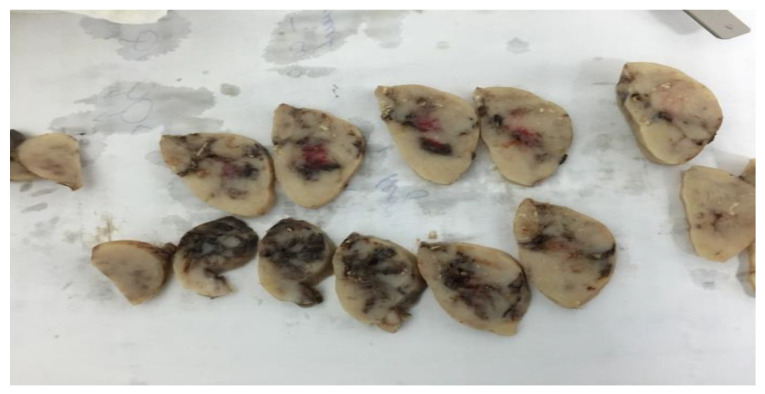
The tumor’s macroscopic features include a brown color, an elastic consistency, and clear calcifications on each part.

Figure 3 shows the macroscopic aspects of the tumor as a nodular formation of 5.6/3.7/3.4 cm, delimited by a thin transparent capsule, brown-gray color on section with small brown areas, and elastic consistency with punctate calcifications.

**Figure 4:**
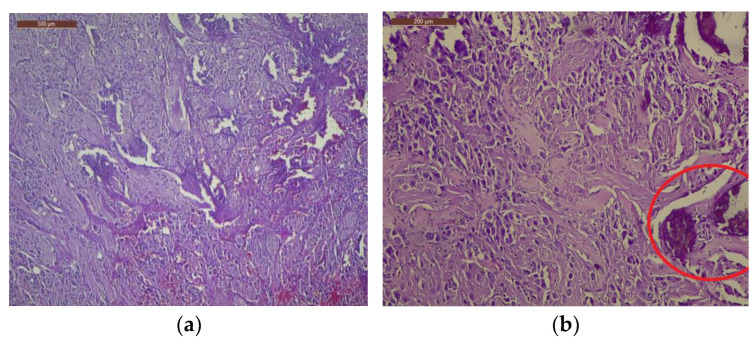
Visualization of tumor cell nuclei characterized by conspicuous enlargement, accompanied by discernible nucleoli and cytoplasm exhibiting a dense eosinophilic nature: (**a**) Within the tumor cell population, minor regions of hemorrhage have been noted, 4× magnification; (**b**) observe the tumor’s growth pattern displaying either lobular or solid arrangements, accompanied by notable instances of dystrophic calcification, 10× magnification. The examination was performed using H&E staining. H score for NSE = 235, NF200 = 230, S100 = 298, GFAP = 288.

The tumor was histologically determined to be an INSS II-localized ganglioneuroblastoma intermixed with a stroma-rich (Shimada Classification) tumor with complete gross excision, positive lymph nodes, and a low MKI (mitosis-karyorrhexis index) of less than 100. The growth pattern of the tumor was solid/lobular. Large, round-to-ovoid nuclei and an abundance of basophilic cytoplasm were features of the tumor cells. A “salt and pepper appearance” with prominent nucleoli and alternate regions of euchromatin and heterochromatin was seen. Other tumor cells were distinguished by rich, eosinophilic cytoplasm, eccentric nuclei, and prominent cell boundaries. The tumor cells contained a small quantity of bleeding and had somewhat calcified dystrophic regions (Figure 4).

**Figure 5:**
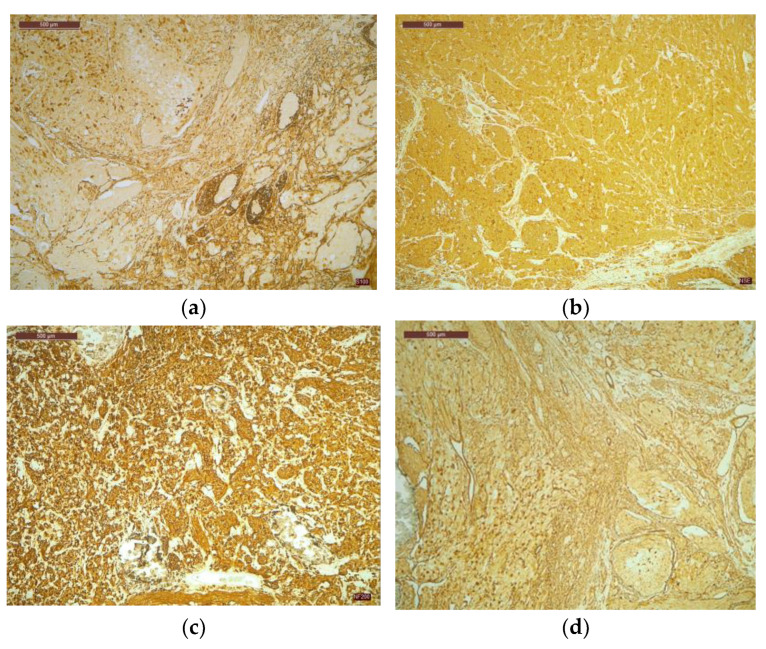
(**a**) Protein S100 demonstrated a robust and positive response in both Schwann cells and ganglion cells; (**b**) tumor cells exhibited a strong NSE expression, characterized by a cytoplasmic pattern of reactivity; (**c**) notably, tumor cells displayed a marked, widespread, and relatively uniform NF 200 expression; (**d**) GFAP demonstrated a moderate to strong positive expression in the ganglion cells. The magnification for the specimen was ×4.

Additional immunohistochemistry testing was conducted using the following panel of markers to confirm the final diagnosis and rule out other lesions with a similar appearance: Chromogranin, Neurofilament (NF 200), Protein S 100 (S100), Glial Fibrillary Acidic Protein (GFAP), Neutron-Specific Enolase (NSE), Synaptophysin, and T-Cell Surface Glycoprotein E2 (CD99). As an internal control marker, vimentin showed a favorable stromal response. The tumor cells responded moderately to strongly to NSE, Synaptophysin, and NF 200. The distribution of the cytoplasmic response pattern was diffuse and heterogeneous. In the ganglion cells and the stroma, immunohistochemistry for S100 and GFAP was highly reactive. S100 was also present in the cytoplasm of Schwann cells. Chromogranin was found to be isolated positively in neuroblasts. Overall, CD99 was rated as unfavorable (Figure 5).

Additional molecular biology analyses were conducted. The status of the N-MYC gene was evaluated using fluorescence in situ hybridization (FISH), yielding a negative result.

The patient experienced a smooth postoperative recovery. Diarrhea subsided, and laboratory findings indicated the restoration of plasma potassium levels to normal. Importantly, there was no recurrence of diarrhea during the three-year follow-up period. The plasma VIP concentration consistently remained at low levels in successive measurements after the surgical procedure.

## 4. Discussions

The first report on VIPoma dates back to 1970. Since then, about six review articles have been published studying the pediatric population. In these articles, 61 pediatric cases were recovered before primary exclusion. However, the incidence of cases in children has not yet been established. Although the prevalence of the pediatric population is unknown, the estimated annual incidence is roughly 1 in 10 million [4,13].

The mean diagnostic age for VIPoma varies widely, from childhood to eighty years, according to various studies [19]. In children, the age of diagnosis is mainly between 2 and 4 years, the lowest reported instance being 2 weeks [20,21]. In terms of gender, in these studies, a predominance of females was found (1:2.46) [4].

The majority of VIPoma cases are single tumors, but about 5% of people with multiple endocrine neoplasia type 1 (MEN1) syndromes also have VIPoma. VIPoma is a rather rare pathology that represents 1–2% of all pancreatic neoplasms. Every year, one in a million people are found to have VIPomas [22].

A functioning pancreatic neuroendocrine tumor (pNET), known as the VIP-secreting tumor, or VIPoma, is extremely rare and life-threatening [23].

PNETs are regarded as a subset of neuroendocrine tumors and have distinctive biology, natural histories, and treatment options. Depending on whether they emit peptide hormones that cause certain hormone-related symptoms, typically in predictable patterns based on tumor subtype, these tumors are categorized as “functional” or “non-functional” [7]. Because they originate in the pancreas, most VIPomas are categorized as functional pNET (islet cell).

VIP is secreted by this particular form of endocrine tumor. In 50 to 75% of patients, it affects the D1 cells of the pancreatic islets of Langerhans, and, at one out of every two cases at the time of diagnosis, lymph node metastases and/or liver are present [21]. Usually, the site of the tumor is in the pancreas, in 80% to 98% of cases, the most commonly occupied being the body and tail of the pancreas, often presenting as a single tumor larger than 2 cm [24].

Chronic diarrhea in childhood is a fairly common pathology and can be the consequence of many gastrointestinal disorders. Four different pathophysiological mechanisms underlie the etiology of chronic diarrhea, and they often overlap: osmotic, secretory, associated with dysmotility, and inflammatory diarrhea. Only a small percentage of these cases involve true secretory diarrhea, which is the only type of diarrhea that lasts through extended fasting.

Low stool osmotic gap (290-calculated stool osmolarity = stool osmotic gap) is a key indicator of secretory diarrhea, whereas high stool osmolarity and ending diarrhea with the removal of the trigger from the meal are specific to osmotic diarrhea. Inflammatory diarrhea is characterized by pathological feces that contain blood, mucus, and bacteria. Diarrhea accompanied by dysmotility, such as toddler diarrhea, is more challenging to diagnose and frequently remains one of exclusion [25].

In most cases, there was a long time between the onset of diarrhea and the diagnosis of the tumor. This is supported by the results of two retrospective studies performed on cases of VIPoma in childhood. Murphy et al. conducted an investigation involving a cohort of six patients, elucidating an average hospitalization duration of four weeks for diagnostic evaluations. Notably, the period between the initial recommendation for hospitalization and the subsequent establishment of a diagnosis exhibited variability, spanning from one to ten months; the mean duration of persistent diarrhea was 5 months, and at the time of diagnosis, five children were severely malnourished [26]. Bourdeaut and colleagues had similar results to Murphy’s in a study of 22 children with VIP-secreting neuroblastic tumors [27].

An early diagnosis of childhood VIPomas has been made possible using a diagnostic algorithm (certain clinical features). Age is the initial clinical trait. Although WDHA has been described in people of various ages, it seems to be more prevalent in babies and young children. The average age of presentation was 21 months in the Iida study from 1980 [26], 18 months in the Bourdeaut study from 2009 [27], and up to 24 months in all cases from the Murphy et al. study from 2000 [28].

Chronic diarrhea is the second symptom. It is reasonable to suspect secretory diarrhea and a VIP secretory tumor when there is persistent watery diarrhea that is resistant to fasting food and total parenteral nutrition over 48–72 hours. Bourdeaut et al. [27] observed that 16 out of 22 patients had diarrhea as a symptom at presentation. The remaining individuals experienced diarrhea following chemotherapy, a condition known as “secondary VIP secretory neuroblastic tumor”. This seems to be a result of VIP release during chemotherapy-induced tumor lysis. Another more tenable explanation holds that chemotherapy concurrently generates weakly differentiated neuroblastic tumors that do not release VIP as well as their capacity to do so [27,28].

The third clinical sign is blood pressure. Blood pressure above the 99th percentile on repeated measurements should raise the suspicion of a neural crest tumor, especially if other signs and symptoms are present. This sign is not always present. For example, Swift et al. [29] presented the case of a child with a neurogenic tumor who had normal blood pressure. However, high blood pressure is well known to be associated with neurogenic tumors in children.

Iida et al. [26], in their study, found that flushing can also suggest the diagnosis (the fourth clinical sign). In the group of patients in their study, flushing was present in 7 of 31 cases. It was found to be an inconsistent and transient sign, however, and may go unnoticed [26]. Finally, according to other authors, two other signs present can be sweating and waddling gait [30].

Werner–Morrison Syndrome is a well-defined clinical and pathological condition that is uncommon but most frequently associated with malignancies that produce VIP [1]. Most pediatric VIPomas are either ganglioneuromas or ganglioneuroblastomas and originate in the neural crest tissue of the sympathetic ganglia or the adrenal medulla. While ganglioneuroblastomas demonstrate varied differentiation and a somewhat uncertain prognosis, ganglioneuromas are well-differentiated and benign tumors [31]. Anywhere sympathetic nerve tissue is present, these tumors may develop. The adrenal glands (35%) and paraspinal retroperitoneal ganglia (30–35%) are the most frequent sites, followed by the posterior mediastinum (20%), head and neck (1–5%), and pelvis (2–3%). Rare sites include the thymus, lung, kidney, and anterior mediastinum [32].

In most pediatric diseases, the microscopic appearance is represented by a neoplasm of neuroectodermal origin composed of a mixture of neuroblasts and ganglion cells in different proportions. The stage of neuroblast development differs between ganglioneuromas, ganglioneuroblastomas, and neuroblastomas.

Ganglioneuromas, which include both Schwann and ganglion cells, are regarded as benign tumors.

In 1915, Robertson defined ganglioneuroblastomas as a transitional tumor of sympathetic cell origin that contained malignant neuroblastomatous and benign ganglioneuromatous element [33].

It is a tumor on the way to differentiation/maturation, but this process is not complete. Microscopic Ganglioneuroblastoma-intermixed (Schwannian stroma-rich) is scattered “residual” microscopic neuroblastomatous foci that come in the form of neuropil pockets containing a variable amount of neuroblastic cells at different stages of maturation and/or ganglion cells. The ganglioneuromatous constituent’s proportion within the microscopically assessed total tumor volume must surpass 50% [34].

Over time, ganglioneuroblastomas have been a challenge for both pathologists and oncologists because there have been no clear and objective boundaries between neuroblastomas and ganglioneuroblastomas and between ganglioneuroblastomas and ganglioneuromas but also because clear guidelines for predicting the tumor biology and clinical prognosis of individuals with ganglioneuroblastomas have not been established. After the update of the WHO 2017 classification system for neuroendocrine tumors (NETs), the histological diagnosis followed this reporting format, which emphasizes a mitotic rate and a Ki-67 index [19]. The International Neuroblastoma Staging System (INSS) and the Shimada system are applicable for prognostics.

The majority of ganglioneuroblastomas are thought to be more aggressive tumors that typically develop in young children, with a mean age of onset of around two years. They are less mature forms made up of neuroblasts and ganglion cells.

In our case, molecular biology tests showed N-MYC negative; this is also found in studies of neuroblastic tumors where most Ganglioneuroblastomas do not show MYCN amplification [35]. The neuromelanin pigment inclusions were not identified in the examined sections, which are documented in the literature as being rare.

This patient presented several specific clinical features from the diagnostic algorithm described above, such as young age at the onset of the disease, chronic watery diarrhea refractory to fasting food and total parenteral nutrition, facial flushing, and sweating. In our case, the blood pressure was normal, although high blood pressure is well known to be associated with neurogenic tumors in children.

Individuals diagnosed with ganglioneuroblastomas generally experience a favorable outlook, given that these tumors have the potential to regress naturally or develop into ganglioneuromas. Regressive changes are observed in approximately 1–2% of all cases, and the underlying reasons for this phenomenon remain unidentified [31].

In the 1960s, an initial hypothesis emerged suggesting that watery diarrhea resulted from the excessive secretion of catecholamines, a well-established phenomenon associated with neural crest tumors [36]. Subsequently, in the 1970s, researchers substantiated that these tumors also produced VIP [37]. VIP, composed of 28 amino acids and with a molecular weight of 3381, belongs to the secretin-glucagon family [38]. In its normal expression, VIP is found in the central nervous system and the neurons of the gastrointestinal, respiratory, and urogenital tracts, playing a role as a neurotransmitter. The overexpression of VIP leads to diarrhea, while the overexpression of VIP receptors fosters the growth of cancer cells. In the gastrointestinal tract, VIP is responsible for relaxing both vascular and nonvascular smooth muscle cells and for triggering the secretion of water and electrolytes. It is released in response to intestinal distention caused by food consumption. VIP acts as a potent inducer of intestinal cAMP production, resulting in the significant release of water and electrolytes, primarily potassium. When endocrine tumors secrete VIP in substantial amounts, patients typically encounter profuse secretory diarrhea, dehydration, flushing, and weight loss [39].

The presented case report indicated an elevation in VIP concentration. Furthermore, hypokalemia was observed. Roughly 90% of children diagnosed with neuroblastoma will exhibit elevated levels of vanillylmandelic and homovanillic acid production. If vanillylmandelic acid and other catecholamine levels fall within the normal range, the likelihood of a neuroblastoma diagnosis in the child is reduced. However, it is important to note that this diagnosis cannot be completely ruled out [40]. Catecholamine production in these tumors is not uniform, leading to variations in hormone and metabolite levels within the urine. Consequently, the tested samples may not consistently show heightened levels [41]. Present predominantly in neuronal and neuroendocrine tissues, neuron-specific enolase (NSE) is a gamma-homodimer with a molecular weight of 78 kD. Aside from erythrocytes, its detectable levels are absent in other tissues [42]. Because of its specificity to certain organs, levels of NSE in the serum or, more commonly, in the cerebrospinal fluid tend to be elevated in conditions leading to neuronal damage. Neural crest-derived tumors also frequently exhibit an increased expression of NSE. Around 70% of small-cell lung carcinoma patients exhibit elevated serum NSE levels upon diagnosis. Other neuroendocrine tumors with frequent NSE expression include carcinoids (in up to 66% of cases), islet cell tumors (usually in less than 40% of cases), and neuroblastoma, although the exact frequency of NSE expression in neuroblastoma remains unknown [43].

In this instance, the 24 h urinary levels of vanillyl mandelic acid and homovanillic acid were within the normal range. However, the serum level of neuron-specific enolase was elevated. This, coupled with the elevated serum levels of VIP, pointed towards the diagnosis of a VIP-producing ganglioneuroblastoma. This diagnosis was subsequently confirmed through histopathological examination and immunohistochemical staining.

Following the International Neuroblastoma Risk Group (INRG) classification [44], this patient was categorized within the low-risk group due to considerations of histological criteria, differentiation level, age, and a negative N-MYC test.

The infant presented in this case report was presumed to have a gastrointestinal disorder, consequently delaying the diagnosis. The patient underwent four weeks of outpatient different investigations, and the interval starting from hospital admission to diagnosis was two weeks. She received empirical trials of treatment for various unproven gastrointestinal disorders. The prompt identification of the diarrhea’s secretory nature might have expedited an early diagnosis, potentially preventing needless investigations and reducing associated morbidity.

While bacterial and viral gastroenteritis can lead to secretion, sustained secretory diarrhea is infrequent. Secretory diarrhea may stem from uncommon hereditary electrolyte transport abnormalities, like congenital chloride diarrhea or congenital sodium diarrhea, yet these disorders typically manifest in early infancy [45]. In this instance, the onset of the symptoms was delayed until 10 months of age, which allowed us to initially rule out the possibility of congenital electrolyte transport problems. In older children, serious gastrointestinal conditions like Crohn’s disease or short bowel syndrome can co-occur with secretory diarrhea. In such circumstances, digestive dysfunction is evident.

In a publication authored by Bourdeaut (2009) [27] involving a cohort of 22 patients, the majority of individuals exhibited weight loss and metabolic disturbances. Within this group, 16 cases displayed gastrointestinal symptoms that preceded the tumor diagnosis; among them, 15 cases exhibited localized lesions characterized by differentiated histology. Among another subset of six patients diagnosed with high-risk neuroendocrine tumors (NETs), episodes of diarrhea manifested during chemotherapy or retinoic acid therapy. Response differentiation to treatment was observed in four instances. Across all cases, exclusive surgical removal of the tumor was effective in managing gastrointestinal symptoms, a parallel observed in our case. Remarkably, 13 children achieved a state of complete remission [27].

A literature review with a case series presentation published by Pai-Jui Yeh in 2020 [4] described data similar to our findings. Particularly, 38 articles containing 61 cases with VIP-secreting tumors were analyzed. The average age at diagnosis was 3.3 years, but it could have been as young as 0.7 years. The median time from the onset of symptoms to the diagnosis was five months. In contrast to other symptoms, including facial flushing, fever, sweating, vomiting, and abdominal pain, all cases started with diarrhea. There were inconsistent reports of hypertension. The three most prevalent laboratory abnormalities were achlorhydria (26.7%), acidosis, and hypokalemia (84.4%). The median plasma VIP level stood at 685 pg/mL, ranging from 200 to 7000 pg/ml. VIP was detected in tumors in 20 cases (44%). Commonly employed imaging techniques encompassed abdominal ultrasonography, CT scans, magnetic resonance imaging (MRI), and angiography. Concerning lesion locations, the majority were tumors outside the pancreas. About 46.7% of cases were situated in the adrenal and suprarenal regions, followed by paravertebral/prevertebral locations (15.6%) and the mediastinum (13.3%). A mere two cases (4.4%) exhibited pancreatic lesions [4].

Concerning histological alterations, in our instance, the neuroblasts did not exhibit the distinctive pseudo-rosette arrangement, and there was an absence of cystic degeneration. The presence of neuromelanin pigment inclusions, which are rarely documented in the literature, was not observed. Ganglioneuroblastomas present a challenging clinical and pathological scenario. Distinguishing morphological and immunohistochemical differences between ganglioneuromas, neuroblastomas, and Schwannomas displaying neuroblastoma-like features is considered a complex diagnostic task. Reaching a definitive diagnosis for these uncommon pathological cases involves a multifaceted process that heavily relies on integrating the patient’s clinical presentation, imaging findings, routine histopathological analysis, and supplementary immunohistochemical assessment of the samples.

## 5. Conclusions

VIPomas frequently manifest with diarrhea as the sole clinical manifestation, necessitating meticulous gastrointestinal evaluations and therapeutic approaches.

This instance depicts a distinctive occurrence of a VIP-secreting ganglioneuroblastoma in an infant, wherein diarrhea emerged as the primary clinical indicator. When faced with a pediatric patient experiencing persistent watery diarrhea of uncertain origin, it is advisable to conduct comprehensive gastrointestinal assessments alongside abdominal ultrasonography and/or CT scans to ascertain the presence of an underlying neoplasm. Morphologically, the examined tumor exhibited distinct characteristics that rendered the differential diagnosis a complex endeavor.

## Data Availability

The data presented in this study are available on request from the corresponding author. The data are not publicly available due to privacy.
